# Level of attitude toward complementary and alternative medicine among Iranian patients with multiple sclerosis

**Published:** 2014

**Authors:** Mohammad Hossein Harirchian, Mohammad Ali Sahraian, Amir Hosseinkhani, Nasibeh Amirzargar

**Affiliations:** 1 Department of Neurology, School of Medicine, Tehran University of Medical Sciences, Tehran, Iran; 2 Department of Neurology, School of Medicine, Tehran University of Medical Sciences, Sina MS Center, Tehran, Iran; 3 Department of Neurology, School of Medicine, Tehran University of Medical Sciences AND Iranian Center of Neurological Research, Tehran, Iran

**Keywords:** Alternative Medicine, Complementary Therapies, Multiple Sclerosis

## Abstract

**Background:**

Multiple sclerosis (MS) is an unpredictable neurological disease leading to severe disability in young adults. The majority of MS patients use complementary and alternative medicine (CAM) as adjunct to conventional therapies. This study aimed to investigate the prevalence of CAM utilization among Iranian patients with MS and their attitude toward the CAM usage.

**Methods:**

A cross-sectional study was conducted on 119 definite MS patients referred to Tehran’s Imam Khomeini and Sina hospitals. A questionnaire was used to examine the association between participants’ health-related factors and usage of CAMs interventions. P value < 0.05 was considered statistically significant.

**Results:**

Among the enrolled patients, 60% of the participants agreed with using CAM, 42% experienced the usage of these treatments; out of whom 41% believed its efficiency and 18% reported exacerbation of symptoms. The mean duration of disease diagnosis and mean time from symptoms onset were both longer in users of CAM (P = 0.001). Most socio-demographic factors had no significant effect on the type of used CAM. However, Yoga was significantly more applied in those with higher degree of education (P = 0.002).

**Conclusion:**

Regarding the widespread use of CAM by Iranian patients with MS, further researches about the safety and efficacy of each treatment on the special outcomes is recommended.

## Introduction

Multiple sclerosis (MS) is an unpredictable neurological disease characterized by chronic course of exacerbation and remission with a wide range of accompanying symptoms, leading to severe disability in young adults and there is no known cure for it.^1–3^ There are more than 40,000 diagnosed MS cases in Iran and the trend is increasing.^4^

Treatments for MS are divided into two categories i.e. disease modifying drugs to control the disease process, and methods to manage symptoms.^5^ Over the last few years, complementary and alternative medicine (CAM) utilization has rapidly grown in the general population and the results from review of the literature showed that MS patients reported 27-100% CAM usage.^1^, ^6^ National Institute of Health defines CAM as “a group of diverse medical and health care systems, practices, and products that are not presently considered to be part of conventional medicine”. Recent guidelines affirmed that MS patients should be informed by findings on the benefits of certain approaches but declared that insufficient evidence is available to make a firmer recommendation.^7^

Many factors influence CAM usage. One of the major reasons is dissatisfaction from currently available treatments and anecdotal reports of CAM's guide.^8^, ^9^ MS patients are facing the fact that their disease cannot be cured by any known conventional medicine and symptomatic treatments have partial effects with a number of adverse reactions.^10^, ^11^ Lack of intimate physician-patient relationship is also a reason for turning to CAM, perhaps because of inadequate information regarding nutrition, exercise and ignorance of spiritual dimensions.^1^, ^6^ Survey results suggest that MS patients desire to use both CAM and conventional medicine to attain a more holistic healthcare approach on body, mind and spirit;^9^, ^12^, ^13^ perhaps attributable to their psychological effects by reducing stress, which is a known factor that exacerbates MS symptoms.^14^, ^15^ In particular, active coping strategies, such as searching for information seem to have positive impact on CAM utilization.^16^ Regarding 50% of MS patients would have depression during the course of the disease; some showed current CAM utilization is related to higher depression scores.^17^ Determination for more personal involvement in the healing process in order to maintain the control over self-healthcare decisions is discussed as another possible reason for using CAM.^18^

Although CAM is widely used by MS patients, there is no scientific evidence-base to support its efficiency.^19^ Knowledge about the prevalence of CAM treatments being used is valuable. Physicians must be aware of the meaning of using alternative modalities on the quality of life of these patients, and provide guidance and monitoring in usage of these therapies to improve outcomes.^17^, ^20^

We aimed to identify the prevalence and frequency of CAM utilization among MS patients referred to our clinic and investigate Iranian patients’ attitude toward the CAM usage and also assess its main components.

## Materials and Methods

This cross-sectional study was conducted on MS patients referred to neurology clinic or ward at Tehran's Imam Khomeini and Sina hospitals. All the patients were eligible to participate in this study for whom the diagnosis of MS was confirmed by neurologist including relapsing remitting or progressive subgroups.

According to the prevalence of using CAM pointed in the literatures, the estimated sample size was 119 patients. They were enrolled by conventional sampling method.

The institutional Ethics Committee approved the study. Written informed consent was obtained from every participant.

All the questionnaires were accomplished face-to-face by the same trained medical student including demographic information such as age, sex, marital status, ethnicity, educational level, duration and type of disease and treatments, family history of MS and 13 questions about previous experience of using CAM and the level of patients’ attitude toward these treatments.

The most common CAM methods among Iranian patients included talisman closing, homeopathy, acupuncture, oxygen therapy, bee stings, electric based methods, warm water, yoga, energy therapy, bloodletting, pray writhing and Kambozia mushroom. Energy therapy proposes that, health can be restored by rebalancing the body's energy field using methods dependent on healer's hands. Bloodletting is based on an ancient medicine in which small quantities of blood are withdrawn from a patient to put humors in proper balance for maintaining the health and curing diseases. Pray writing is inscribing religious word on different things with this belief that it could resolve their illnesses and problems. Some patients place credence in a traditional drink from china which use Kambozia mushroom for fermentation of tea with sugar that excrete undesirable body materials.

Results were presented as mean ± standard deviation (SD) for quantitative variables which were compared with t test or Mann-Whitney U test. Categorical variables were compared using chi-square test or Fisher's exact test which were used to examine the association between participants’ health-related factors and usage of CAM. Questionnaire responses were analyzed with the SPSS for Windows 16.0 (SPSS Inc., Chicago, IL, USA). P-values < 0.05 were considered statistically significant.

## Results

Among 119 enrolled patients, 77.3% were females and 68.9% were married. Respondents had a median age of 35.4 ± 9.2 years. Regarding educational state, 40.3% had university degree. The mean duration of disease diagnosis was 6.1 ± 4.6 years, while the mean time of symptoms onset was 8.1 ± 5.2 years. Family history of MS in first relatives was expressed by 3.4% and in second relatives by 12.6%. The patients were from different ethnicities. Overall, 51 patients (42.85%) experienced the use of CAM; 60.5% of participants were agreed with using these therapeutics. In addition, 41.17% found these methods to be effective, 41.17% believed to be ineffective, and 17.64% expressed that these approaches led to a worsening of symptoms. More details are illustrated in Table 1.

**Table 1:** Frequency of opinion, usage and perception of efficacy for complementary and alternative medicine (CAM) products amongst MS patients

	Patients’ opinion about the Use of CAM (%)			Use of CAM (%)		Effect of using CAM on patients’ symptoms (%)		

Methods	Agree	Disagree	No opinion	Yes	No	Improving	Worsening	Ineffective
Energy therapy	34.5	22.7	42.9	28.6	71.4	35.3	2.9	55.9
Homeopathy	9.2	7.6	83.2	11.8	88.2	50.0	7.1	35.7
Acupuncture	27.7	25.2	47.1	10.1	89.9	8.3	16.7	75.0
Bloodletting	18.5	45.4	36.1	13.4	86.6	31.3	6.3	62.5
Oxygen therapy	11.8	11.8	75.6	1.7	96.6	100	0.0	0.0
Bee stings	31.9	27.7	40.3	5.9	94.1	57.1	0.0	28.6
Electric based methods	12.6	38.7	48.7	5.0	95.0	50.0	16.7	33.3
Talisman closing	16.8	75.6	7.6	7.6	92.4	22.2	22.2	44.4
Pray writhing	25.2	63.9	10.9	21.8	78.2	38.5	11.5	42.3
Kambozia mushroom	7.6	5.0	87.4	3.4	96.6	0.0	0.0	100
Warm water	20.2	67.2	12.6	15.1	83.2	27.8	50.0	16.7
Yoga	55.5	6.7	37.8	16.8	83.2	85.0	0.0	10.0

There was no statistical significant difference between the mean age of users and nonusers of CAM (P = 0.78). However, the mean duration of disease diagnosis and mean time from symptoms onset were both longer in users (P = 0.001). Patients who used acupuncture were significantly older than nonusers (P = 0.01). The significant longest duration of disease diagnosis and symptoms onset were revealed in the groups used bloodletting (P = 0.01), homeopathy (P = 0.03) and energy therapy (P = 0.001) (Table 2). Age and marital status had no significant effect on the type of used CAM (P = 0.12 and P = 0.28). Only using Kambozia mushroom was significantly higher in men than in women (P = 0.03) (Table 3). No statistical significant association was observed between the type of CAM and educational level. The most common applied methods in high-school graduates group were pray writing, warm water therapy, energy therapy, and bloodletting; in diploma group were energy therapy, warm water therapy, and pray writing, and in college degree group were energy therapy, yoga, and pray writing. Only yoga was significantly more applied in those with university degree compared to other educational level groups (P = 0.002). No association was found between the presence of MS disease in the first and second degrees of relatives and the type of these therapies usage. Applying yoga, bloodletting, and acupuncture was more observed in the patients with affected first relatives and the use of warm water, yoga, and pray writing was more found in those with affected second relatives. Among different ethnicities, only talisman closing had significant distinction (P = 0.03).

**Table 2:** Mean age, mean duration of disease diagnosis and mean time from symptoms onset amongst each complementary and alternative medicine (CAM) treatment modalities used by people with MS

	Mean age (year)			Mean duration of disease diagnosis (year)			Mean time from symptoms onset (year)		

Usage of CAM	Yes	No	P	Yes	No	P	Yes	No	P
Energy therapy	36.4 ± 10.2	35.0 ± 8.8	0.43	8.2 ± 4.5	5.2 ± 4.4	0.01	10.1 ± 5.1	7.3 ± 5.0	0.01
Homeopathy	36.4 ± 10.1	35.2 ± 9.1	0.66	8.6 ± 4.0	5.8 ± 4.6	0.03	10.7 ± 5.0	7.7 ± 5.1	0.04
Acupuncture	41.7 ± 8.8	34.7 ± 9.0	0.01	5.9 ± 3.2	6.1 ± 4.7	0.86	8.0 ± 2.1	8.1 ± 5.4	0.95
Bloodletting	39.1 ± 9.2	34.8 ± 9.1	0.08	8.8 ± 6.0	5.7 ± 4.2	0.01	11.5 ± 6.6	7.5 ± 4.8	< 0.01
Oxygen therapy	29.0 ± 0.0	35.4 ± 9.3	0.01	10.0 ± 0.0	6.1 ± 4.6	0.24	11.0 ± 0.0	8.1 ± 5.2	0.44
Bee stings	33.8 ± 4.7	35.5 ± 9.4	0.42	7.8 ± 3.0	6.0 ± 4.7	0.31	13.2 ± 5.0	7.8 ± 5.1	0.01
Electric based methods	35.5 ± 12.8	35.4 ± 9.0	0.98	8.5 ± 9.6	6.0 ± 4.2	0.55	9.5 ± 9.3	8.0 ± 4.9	0.72
Talisman closing	32.8 ± 6.9	35.6 ± 9.3	0.39	6.9 ± 3.3	6.0 ± 4.7	0.58	8.6 ± 3.4	8.0 ± 5.3	0.74
Pray writhing	36.5 ± 9.5	35.1 ± 9.1	0.48	6.5 ± 4.4	6.0 ± 4.6	0.58	8.8 ± 5.5	7.5 ± 5.1	0.40
Kambozia mushroom	35.0 ± 9.8	35.4 ± 9.2	0.92	9.2 ± 3.5	6.0 ± 4.6	0.17	10.0 ± 4	8.0 ± 5.2	0.46
Warm water	37.5 ± 7.9	34.9 ± 9.4	0.29	6.6 ± 3.1	5.9 ± 4.7	0.40	8.5 ± 4.9	7.9 ± 5.2	0.68
Yoga	32.0 ± 9.2	36.1 ± 9.1	0.06	6.1 ± 3.9	6.1 ± 4.7	0.96	8.3 ± 3.6	8.0 ± 5.5	0.83

**Table 3:** Frequency of complementary and alternative medicine (CAM) treatment modalities used by multiple sclerosis patients according to gender and marital status

	Male (%)		Female (%)			Single (%)		Married (%)		

Use of CAM	Yes	No	Yes	No	P	Yes	No	Yes	No	P
Energy therapy	37	63	26.1	73.9	0.26	27.0	73.0	29.3	70.7	0.80
Homeopathy	18.5	81.5	9.8	90.2	0.30	10.8	89.2	12.2	87.8	1.00
Acupuncture	18.5	81.5	7.6	92.4	0.14	2.7	97.3	13.4	86.6	0.07
Bloodletting	22.2	77.8	10.9	89.1	0.19	16.2	83.8	12.2	87.8	0.55
Oxygen therapy	0.0	100	2.2	97.8	1.00	0.0	100	2.5	97.5	1.00
Bee stings	7.4	92.6	5.4	94.6	0.65	2.7	97.3	7.3	92.7	0.43
Electric based methods	7.4	92.6	4.3	95.7	0.61	2.7	97.3	6.1	93.9	0.66
Talisman closing	7.4	92.6	7.6	92.4	1.00	8.1	91.9	7.3	92.7	0.88
Pray writhing	18.5	81.5	22.8	77.2	0.63	18.9	81.1	23.2	76.8	0.60
Kambozia mushroom	11.1	88.9	1.1	98.9	0.03	2.7	97.3	3.7	96.3	1.00
Warm water	11.1	88.9	16.7	83.3	0.76	8.1	91.9	18.8	81.3	0.13
Yoga	29.6	70.4	13.0	87.0	0.07	18.9	81.1	15.9	84.1	0.67

## Discussion

Investigations showed wide spread utilization of CAM despite a limited evidence-base among MS patients.^21^ Up to 80% of MS patients used CAM at one point during their disease.^22^ In our study, 42.85% of cases experienced CAM usage. High agreement of the participants for using CAM (60.5%) was considerable and emphasizes its importance in patients’ priorities.

Some factors are predictive of CAM usage. Most studies examining associations with demographics found a tendency for users to be middle-aged females, most likely reflects the gender distribution of the condition.^23^ Compatible with Germany study,^14^ in our survey although the majority of patients were women, no statistical significant difference was indicated between the mean age, gender and marital status of users and nonusers of CAM. However, Kambozia mushroom was more used by men than women might be due to the availability of this material for men.

Investigations identified being highly educated, having white-collar job and those with higher income level were more likely to utilize CAM.^5^, ^24^, ^25^ The results of the present study showed no association between the educational levels and use or type of CAM selection. Inconsistency with previous observations seems to be the influence of the cultural approaches in our country. Moreover, lack of complementary and alternative therapies as well as absence of accredited center for providing these services may be further causes for lesser interest for using these methods in educated subgroups. In this study, the only statistical significant difference was seen in the usage of yoga that could be due to expensiveness or lack of awareness about the existence of such therapies among people with lower educational levels.

The usage of CAM is also influenced by regional and cultural habits.^25^ For example, the rate of prayer to improve MS symptoms in the United States is consistently reported much higher than the Europe.^22^ Ben-Arye et al. also identified women of Arab descent are less likely to use CAM.^26^ Our study showed only talisman closing had significant distinction between different ethnicities (P = 0.03).

Conventional healthcare providers, friends, family and caregivers were the most frequently reported CAM information sources.^23^ Because of low amount of MS report in the first and second relatives of our patients, judgment about their influence on selecting CAM seems to need more investigations.

Confirming findings of previous studies,^19^, ^22^, ^24^ our survey showed that longer MS duration is a predisposing factor for the usage of CAM. Longer living with disease might be enough to obtain necessary information toward MS and its related treatment variations, especially CAM.

The severity of the disease may also affect the CAM application.^14^, ^19^ Some studies emphasize that physical wellbeing, chronic illness and pain, higher disability and poorer health status cause higher frequency of CAM use.^13^, ^24^ It seems that MS patients are turning towards CAM when the disease proceeds and conventional medication becomes less effective.^6^ However, some researchers discovered that disease severity had no significant influence on the usage of CAMs interventions in MS.^23^, ^27^ Heterogeneous results in various literatures can probably be due to differing definitions for CAM, diverse selection criteria, small sample sizes and differences in the time periods of use.^25^

In the present survey, approximately 41% of the patients believed that CAM improved their symptoms and 17.6% expressed that the usage of these methods exacerbated their disease severity. All the findings obtained based on patients’ personal opinions which need objective or scientific documents. There appeared to be no association between percentage used and perceived efficacy for each CAM. Interestingly, although high negative attitude was found toward pray writing among MS patients, the usage of this method was high which seemed to be due to its availability among different community subgroups.

In the surveys reviewed,^1^ the most frequently used CAM therapies included massage therapy, acupuncture, chiropractic, vitamins/herbs and nutrition. In the present study, the most common used therapies included energy therapy, pray writing and yoga. The tendency toward the use of these methods is widely varied in different populations with discrepant cultures, religions, and beliefs which should be evaluated in future widespread investigations besides the effect of mental status on patient's attitude.

Potential limitations of this study included the retrospective nature of data collection and therefore recall bias and low information of patients which required comprehensive explanation of the questions.

The most mentioned benefits for the most used therapies in literature were positive effects on enhancing relaxation, reducing specific physical symptoms such as pain, spasm and fatigue, progression lagging, inducing general well-being and improving sleep, muscle strength and mobility.^28^

There is cause for concern when considering possible side effects of CAM including contamination, potential conflicts between CAM and established therapies of proven efficacy, and possible psychological and economic consequences.^25^, ^29^

## Conclusion

The results of the present study delineate that approximately 60% of our MS patients had tendency toward usage CAM treatment and almost half of them experienced its usage. According to widespread utilization and the importance of these treatments for individuals with MS, physicians should intensify their efforts to consult individuals adjustable to their needs for improving outcomes. Moreover, there is a necessity for well-designed clinical trials assessing the safety and efficacy of each treatment on the special outcomes in the MS population to differentiate between placebo, psychological and physical effects.
